# Role of Murine Complement Component C5 in Acute *in Vivo* Infection by Pathogenic *Leptospira interrogans*

**DOI:** 10.3389/fcimb.2018.00063

**Published:** 2018-03-08

**Authors:** Íris A. de Castro, Lorena Bavia, Tatiana R. Fraga, Mariane T. Amano, Leandro C. D. Breda, Adriana P. Granados-Martinez, Ana M. G. da Silva, Silvio A. Vasconcellos, Lourdes Isaac

**Affiliations:** ^1^Laboratory of Complement, Department of Immunology, University of São Paulo, São Paulo, Brazil; ^2^Institute of Tropical Medicine, University of São Paulo, São Paulo, Brazil; ^3^Faculty of Veterinary Medicine, University of São Paulo, São Paulo, Brazil

**Keywords:** Complement System, C5, *Leptospira*, leptospirosis, murine model, inflammation

## Abstract

Leptospirosis is considered one of the most important zoonosis worldwide. The activation of the Complement System is important to control dissemination of several pathogens in the host. Only a few studies have employed murine models to investigate leptospiral infection and our aim in this work was to investigate the role of murine C5 during *in vivo* infection, comparing wild type C57BL/6 (B6 C5^+/+^) and congenic C57BL/6 (B6 C5^−/−^, C5 deficient) mice during the first days of infection. All animals from both groups survived for at least 8 days post-infection with pathogenic *Leptospira interrogans* serovar Kennewicki strain Fromm (LPF). At the third day of infection, we observed greater numbers of LPF in the liver of B6 C5^−/−^ mice when compared to B6 C5^+/+^ mice. Later, on the sixth day of infection, the LPF population fell to undetectable levels in the livers of both groups of mice. On the third day, the inflammatory score was higher in the liver of B6 C5^+/+^ mice than in B6 C5^−/−^ mice, and returned to normal on the sixth day of infection in both groups. No significant histopathological differences were observed in the lung, kidney and spleen from both infected B6 C5^+/+^ than B6 C5^−/−^ mice. Likewise, the total number of circulating leukocytes was not affected by the absence of C5. The liver levels of IL-10 on the sixth day of infection was lower in the absence of C5 when compared to wild type mice. No significant differences were observed in the levels of several inflammatory cytokines when B6 C5^+/+^ and B6 C5^−/−^ were compared. In conclusion, C5 may contribute to the direct killing of LPF in the first days of infection in C57BL/6 mice. On the other hand, other effector immune mechanisms probably compensate Complement impairment since the mice survival was not affected by the absence of C5 and its activated fragments, at least in the early stage of this infection.

## Introduction

Leptospirosis is an emerging disease worldwide which affects approximately one million patients each year (Costa et al., 2015), mainly in developing countries with tropical and subtropical climates and underdeveloped waste and sewage management systems. For some patients, this disease can be asymptomatic, while others may present symptoms ranging from a mild infection to the development of fever, jaundice, liver and kidney failure and lung hemorrhage, resulting in fatality rates higher than 5–10% (Torgerson et al., 2015). Infection results from the contact of injured skin or mucosa with soil and/or water contaminated with leptospires released by the urine of infected animals (Ko et al., 2009). Rodents are considered asymptomatic to this pathogen and they represent the main transmission source especially in urban centers (Adler, 2015).

Since *Leptospira* spp. is considered an extracellular pathogen, the activation of the Complement System (CS), phagocytosis and production of specific antibodies play an important role in controlling this infection (reviewed by Fraga et al., 2016). The CS is necessary to control proliferation and dissemination of several microorganisms in the host which is clearly confirmed by the higher susceptibility to infections observed in C5 deficient patients (Aguilar-Ramirez et al., 2009) or in patients deficient of other CS proteins (Macedo and Isaac, 2016). CS can be activated by the Classic, Alternative and/or Lectin Pathways, and all three converge to a common terminal activation pathway which leads to lysis caused by the formation of the membrane attack complex (MAC) on the microorganism surface. The terminal pathway depends on the formation of C5 convertase enzymes which cleave C5 in two fragments. C5a, the smaller fragment, is an important anaphylatoxin involved in mast cell and basophil degranulation which releases histamine and other inflammatory mediators like prostaglandins and leukotrienes (Guo and Ward, 2005). C5a is also a well-known chemoattractant factor for neutrophils, monocytes and eosinophils during acute inflammation. C5b, the larger fragment, is the first to participate in MAC (C5b6789_n_) formation (Podack et al., 1984; Serna et al., 2016). Besides contributing to control systemic or local infection, the inflammatory properties observed during C5 activation and the participation of receptors such as C5aR1 may be responsible for local tissue damage (Ward, 2010). The ability to induce cellular lysis and the synergistic interactions with other immune mechanisms highlight the importance of the CS in mounting a robust immune response.

Nonpathogenic leptospires are rapidly killed *in vitro* after CS activation while pathogenic species such as *Leptospira interrogans* serovar Kennewicki strain Fromm (LPF) are resistant. LPF immune evasion mechanisms include: (i) binding to host CS regulatory proteins Factor H (Meri et al., 2005), C4b binding protein (Barbosa et al., 2009, 2010; Breda et al., 2015) and vitronectin (da Silva et al., 2015); (ii) binding to host proteases such as plasminogen (Vieira et al., 2010, 2011; Castiblanco-Valencia et al., 2016) which once converted to its active form, plasmin, may cleave CS proteins; and (iii) secretion of leptospiral proteases that cleave Complement proteins (Fraga et al., 2014; Amamura et al., 2017).

Even though mice are considered asymptomatic to *Leptospira* infection, the possibility of using congenic mouse models allows researchers to investigate in more depth important questions related to the pathophysiology of the immune responses. In addition, mice are considered good models to study sub-lethal infection and chronic colonization often observed in leptospirosis patients (reviewed by Gomes-Solecki et al., 2017). To date, only one study has investigated the importance of the CS during infection by *Leptospira* spp. in a murine model. Ferrer et al. (2014) studied the relevance of decay accelerating factor (DAF, CD55) at 14 and 90 days after infection. DAF is an important regulatory membrane protein that protects host cells from autologous CS activation by binding to membrane-bound C3b and inhibiting the formation and accelerating the decay of C3-convertases and C5-convertases (Nicholson-Weller et al., 1981, 1983). However, differently from other regulatory proteins like Factor H and Factor I, DAF does not modulate serum levels of C3 and is not related directly to the elimination of microorganisms. Ferrer et al. (2014) observed that C57BL/6J Daf l^−/−^ mice infected with *L. interrogans* serovar Copenhageni presented higher numbers of leptospires in the kidney 14 days post-infection when compared to wild type mice and the lack of DAF was associated with chronic nephritis and renal fibrosis. These symptoms are probably related to persistent injury caused by uncontrolled CS activation on proximal renal tubules.

To evaluate the importance of the component C5 in the control of *in vivo* leptospiral infection, we infected C57BL/6 (B6) wild type (B6 C5^+/+^) and congenic C57BL/6 C5 deficient mice (B6 C5^−/−^) with pathogenic *L. interrogans* serovar Kennewick type Pomona Fromm and analyzed several aspects of the immune response during the first days of infection.

## Materials and methods

### *Leptospira* cultures

Pathogenic *L. interrogans* serovar Kennewicki, strain Pomona Fromm (LPF) and saprophytic *L. biflexa* strain Patoc I (Patoc) were obtained from the Laboratory of Bacterial Zoonosis at the Faculty of Veterinary Medicine and Animal Science of the University of São Paulo. Leptospires were kept under aerobic conditions at 29°C for 5–7 days in Ellinghausen McCullough Johnson and Harris culture medium (EMJH) supplemented with 10% inactivated rabbit serum, L-asparagine (0.015%), sodium pyruvate (0.001%), calcium chloride (0.001%), magnesium chloride (0.001%), peptone (0.03%), and meat extract (0.02%).

### Mice infection with LPF

We used C57BL/6 C5 normal (B6 C5^+/+^) mice and the corresponding congenic C57BL/6 C5 deficient (B6 C5^−/−^) strain, generated in our laboratory (Bavia et al., 2014). We also included another C5 deficient (A/J) mouse strain to evaluate the survival during LPF infection. All mice were 4–5 weeks old males obtained from the Animal Facility of the Department of Immunology, Institute of Biomedical Sciences from the University of São Paulo. Male mice were used to avoid interference by sexual hormones and since they present higher levels of Complement System activity and higher serum levels of C6 and C9 from the terminal pathway (Kotimaa et al., 2016). Mice were intraperitoneally infected with 1.5 × 10^8^ LPF in phosphate buffered saline pH 7.4 (PBS) and euthanized on the third or sixth days after infection. Control groups were inoculated with sterile PBS and euthanized 6 days post inoculation. We selected these days of infection based on previous work (da Silva et al., 2012). Mice were previously anesthetized with ketamine and xylazine (100 and 10 mg/kg, respectively) before manipulation. Hamsters, a susceptible experimental animal, were used to confirm the virulence by infecting them with LPF culture. Three to five days post-inoculation of LPF, infected hamster presented jaundice, photo sensibility, uveitis, weight loss and prostration. This work was carried out as approved by the Ethics Committee on Animal Experimentation (Certificate 061/10/CEEA). The number of mice used in each experiment is indicated in each figure legend.

### DNA extraction from liver and *Leptospira* DNA quantification by qPCR

Total DNA was extracted from 20 to 25 mg of liver from LPF-infected or control (PBS) mice using the Illustra Tissue & Cells Genomic Prep Mini Spin kit (GE Healthcare, Little Chalfont, Buckinghamshire, UK) following the manufacturer's instructions. DNA concentration and purity were measured using a Nanodrop nd-1000 spectrophotometer (Thermo Fisher Scientific).

To determine the leptospiral load in the liver from infected mice, we employed quantitative PCR (qPCR) using 96 well microtiter plates (Life Technologies). A concentration of liver DNA was adjusted to 50 ng/μL. The standard curve was prepared using DNA extracted from 10-fold dilutions from 10^8^ to 10^1^ heat-killed *L. interrogans*. We used 1 μL for the standard curve or of samples followed by 20 pmol/μL of both primers complementary to the *Leptospira 16S rRNA* gene (forward primer: 5′-TAGTGAACGGGATAGATAC-3′; reverse primer 5′-GGTCTACTT AATCCGTTAGG-3′) and 10 μL SYBR Green master mix (Life Technologies) in a final volume of 20 μL. Samples were amplified in a thermocycler Step One Plus Real-Time PCR System (Applied Biosystems, Foster City, CA, USA) with the following program: initial denaturation at 95°C for 10 min, followed by 40 cycles of 95°C for 15 s, 60°C for 1 min, followed by two cycles of 95°C for 15 s and 60°C for 1 min, and a final step at from 0.5 to 95° C (ramp) for 15 s.

### Histopathological and immunochemical analyses

Liver, kidney, lung and spleen samples were fixed in formalin solution (3.7% formaldehyde in PBS pH 7.4). Microscopic slides were prepared with 5 μm tissue sections stained with hematoxylin-eosin (HE). To quantify the histopathological alterations in the liver, we considered the following criteria: (a) sinusoidal hypercellularity and presence of leucocyte infiltrates; (b) presence of mitotic cells; (c) hepatocyte destrabecullation; and (d) cell necrosis. Liver sections with only one of the above criteria were classified with a score of 1; with two of above criteria, given a score of 2; with three criteria, a score of 3 and with all the above criteria, a score of 4. To quantify the histopathological alterations in the lung, we considered the following criteria: (a) presence of nodular interstitial pneumonitis (IP) was classified with a score of 1; (b) presence of diffuse IP was classified with a score of 2. To quantify the histopathological alterations in the spleen, we considered the following criteria: (a) presence of perifollicular hyperplasia was classified with a score of 1; (b) presence of central follicular hyperplasia was classified with a score of 2; (c) presence of both perifollicular and central follicular hyperplasia was classified with a score of 3.

Immunohistochemistry analyses were performed to assess the presence of leptospiral antigens in the organs. Liver, kidney, lung and spleen sections were deparaffinized and rehydrated. Tissue sections were then incubated with Target Retrieval solution (DAKO S1699) heated using a steamer to unmask antigen(s). The presence of endogenous peroxidase was blocked by incubation with 3% H_2_O_2_ for 20 min at room temperature. Preparations were then incubated for 18 h at 4°C with rabbit polyclonal anti-*Leptospira* antibodies diluted at 1:19.000 in PBS supplemented with 0.1% BSA. After several washes with PBS, the tissue sections were incubated with secondary EnVision + System HRP labeled polymer anti-rabbit IgG (DAKO K 4002) for 30 min at room temperature. The slides were washed again and incubated with the chromogen Diamino-benzidine (DAB, Sigma Chemical Co. USA) in the presence of 2% H_2_O_2_ (10 vol.) for 5 min. After this procedure, the tissue sections were stained with hematoxylin.

### Biochemical assays

Hepatic damage was indirectly evaluated by measuring alanine transaminase and aspartate transaminase (AST) (Bioclin Quibasa, Belo Horizonte, MG, Brazil) serum concentrations, while kidney function was evaluated by urea) and uric acid (Bioclin Quibasa, Belo Horizonte, MG, Brazil) serum levels as described by Bavia et al. (2014).

### Blood leukocyte counting

Fresh blood samples were obtained from orbital venous plexus with heparinized glass capillary tubes from anesthetized mice. Samples were diluted in Türk solution (4.76 mM acetic acid, 6.25 μM methylene blue) and total peripheral blood leukocytes were counted in a Neubauer chamber.

### Cytokine measurements

Liver levels of tumor necrosis factor (TNF)-α, interleukin (IL)-1β, IL-6, IL-10, IL-12p40, and IL-12p70 were determined by ELISA as described in Bavia et al. (2015). Serum levels of IL-6, IL-10, monocyte chemoattractant protein-1 (MCP-1), interferon-γ (IFN-γ), TNF-α, and IL-12p70 were determined using the Inflammation CBA kit (BD Bioscience, Franklin Lakes, New Jersey, United States), according to the manufacturer's instructions. Data acquisition was performed using a FACS Canto II flow cytometer and data analysis was performed using FCAP Array™ v3.0.1 Software (both from BD Bioscience, Franklin Lakes, New Jersey, United States).

### Leptospires killing assay

LPF and Patoc cultures were centrifuged at 2,800 × *g* at 21°C for 20 min, resuspended in PBS and counted in a Petroff-Hausser chamber. A total of 1 x 10^8^ leptospires was incubated for 2 h at 37°C with B6 C5^+/+^ or B6 C5^−/−^ serum (40% serum and 60% PBS, 200 μL). Normal human serum (NHS) was used as a positive control (Barbosa et al., 2009). Viable bacteria were counted using dark-field microscopy. To inactivate the CS in mice or human serum, they were previously heated at 56°C/30 min. The number of viable leptospires incubated with heat-inactivated serum was considered 100% survival.

### Statistical analysis

Leptospire killing assay was plotted considering the mean and standard deviation and analyzed by Mann-Whitney test. The other results were plotted with the mean and standard error for each group and submitted to ANOVA two-way with Tukey post-test. All analyses considered a significance level of at least 95% (*p* < 0.05).

## Results

### LPF is detected mainly in the liver in the early days of infection

**Figure 1:**
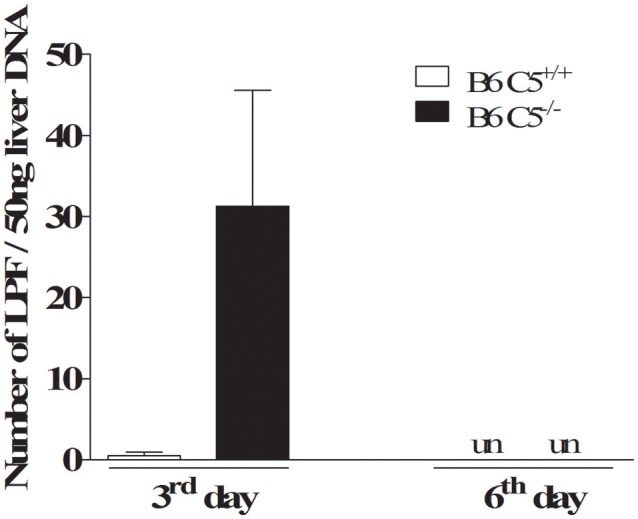
Acute infection with leptospires in C5 deficient mice. Relative number of leptospires in mouse liver was quantified by qPCR. Infected B6 C5^+/+^ and B6 C5^−/−^ mice were euthanized after 3 and 6 days post-infection. Liver DNA was extracted and the relative number of leptospires was determined by qPCR after amplification of *16S rRNA* gene. un: undetectable.

On the third day post-infection, C5 deficient mice (B6 C5^−/−^) were observed to carry a higher number of LPF in the liver when compared to C5-sufficient mice (B6 C5^+/+^). However, on the sixth day post-infection, the presence of LPF was undetectable in both mice strains by qPCR (Figure 1). The presence of LPF antigens in the liver was also investigated by immunohistochemical analysis, but no differences were observed between B6 C5^+/+^ or B6 C5^−/−^ mice (Supplementary Figure 1). The survival of LPF infected mice was independent of C5 since all B6 C5^+/+^ and B6 C5^−/−^ mice survived when monitored up to eight days of infection with LPF. In addition, we used another C5 deficient mouse strain (A/J) with 10^3^, 10^5^, 10^7^, and 10^9^ LPF (minimal of 5 mice for each inoculum). Again, all mice survived up to 21 days of infection.

### C5 triggers more inflammation in the liver during early infection with LPF

**Figure 2:**
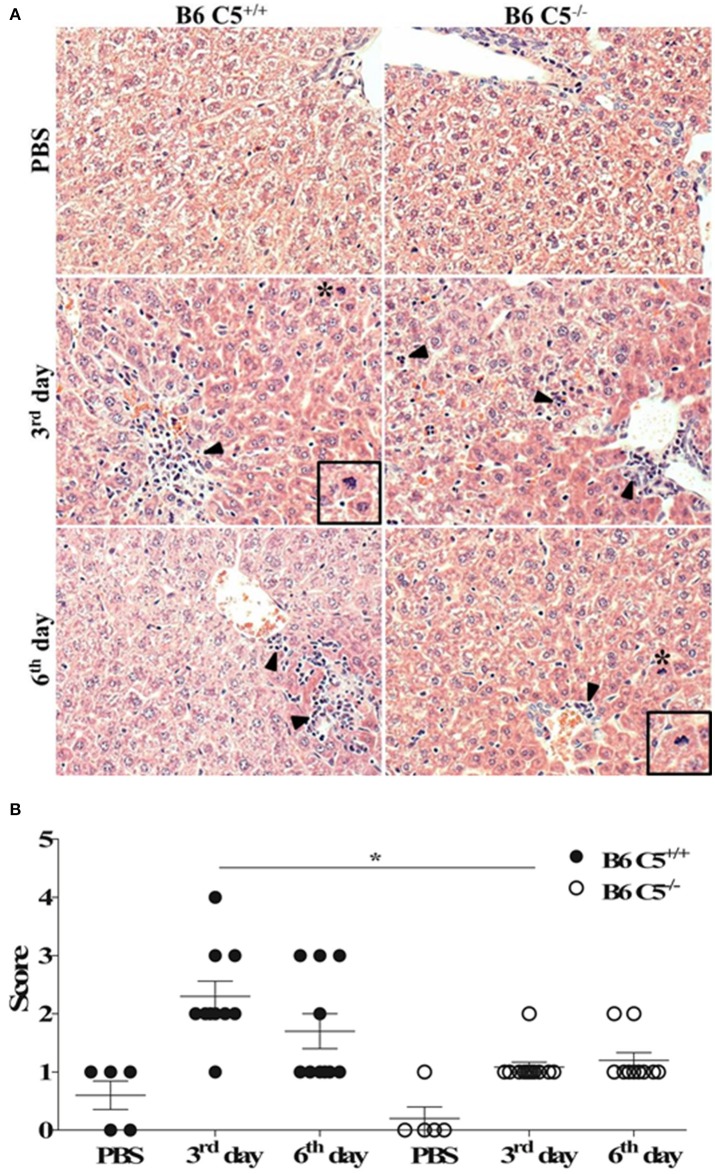
Liver histopathological analyses in LPF infected mice. Mice were inoculated i/p with 1.5 × 10^8^ LPF or only PBS and then euthanized on the third or the sixth day post-infection (*n* ≥ 5). **(A)** Liver sections (3–5 μm) were stained with (HE) and evaluated at 200x magnification. Arrowheads indicate leukocyte infiltrates in the portal space and in the hepatic sinusoids. Asterisks indicate mitotic cells. Inset: mitotic cells in larger magnification. **(B)** Total scores of hepatic lesions. The significant difference (*p* < 0.05) is represented by ^*^when B6 C5^+/+^ were compared to B6 C5^−/−^ mice.

Since C5a and its receptor C5aR1 are clearly associated with liver inflammation, tissue injury and regeneration (Strey et al., 2003; Markiewski et al., 2004, 2009), we compared livers from LPF-infected B6 C5^+/+^ and B6 C5^−/−^ mice. As illustrated in Figure 2A, leukocyte infiltration was observed on the third and sixth days of infection around the portal spaces and within the hepatic sinusoids. This infiltration was composed mainly of mononuclear cells and was more evident in the presence of C5 on the third day of infection. Mitotic cells were also present in B6 C5^+/+^ mice on the third and sixth day, while in B6 C5^−/−^ mitotic cells were found only on the sixth day. These observations suggest an intense inflammatory response accompanied by hepatocellular lesions by hepatocyte proliferation primarily in B6 C5^+/+^ mice (Figure 2A). These hepatic changes were significantly more intense in B6 C5^+/+^ mice than in B6 C5^−/−^ on the third day of infection. On the sixth day of infection, C5 deficient mice continued to present fewer lesions than B6 C5^+/+^ mice but no significant differences in the scores were observed between them (Figure 2B). To monitor liver damage, levels of ALT and AST enzymes were determined in the serum and no significant differences were observed in B6 C5^−/−^ and B6 C5^+/+^ mice (Supplementary Figure 2).

### Lung and kidney of LPF infected mice

During human leptospirosis, lung and kidney may also be affected (Ko et al., 2009). Although LPF antigens were practically undetectable by immunohistochemical analysis in lung and kidney of both B6 C5^−/−^ and B6 C5^+/+^ mice on the third and sixth days post-infection (data not shown), in our model, LPF provoked lesions in lung, characterized by thickening of the alveolar septa accompanied by lymphocyte infiltration (Supplementary Figure 3A). However, no significant differences were observed between B6 C5^+/+^ and B6 C5^−/−^ infected mice (Supplementary Figure 3B). Moreover, no lesions were observed in the kidneys of B6 C5^+/+^ and B6 C5^−/−^ LPF infected mice (Supplementary Figure 4A). In addition, the serum concentrations of urea and uric acid were measured and both were altered at the sixth day of infection. However, these differences were not C5 dependent (Supplementary Figure 4B).

### Blood leukocyte analysis

**Figure 3:**
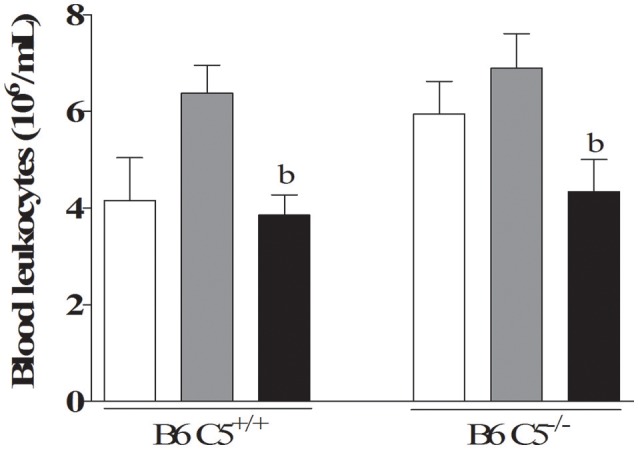
Circulating leukocytes in LPF infected mice. Mice were inoculated i/p with 1.5 × 10^8^ LPF (*n* ≥ 5) and the total number of leukocytes was determined after 3 and 6 days post-infection using a hemocytometer chamber. b represents significant difference between the third and sixth days of infection.

The total number of circulating leukocytes was determined in the blood of both B6 C5^+/+^ and B6 C5^−/−^ mice infected and non-infected with LPF. This number significantly decreased on the sixth day when compared to the third day of infection in both mouse strains infected with LPF (Figure 3). No significant difference could be attributed to the presence of C5 either on the third or the sixth days of infection.

### Spleen modifications during LPF infection

**Figure 4:**
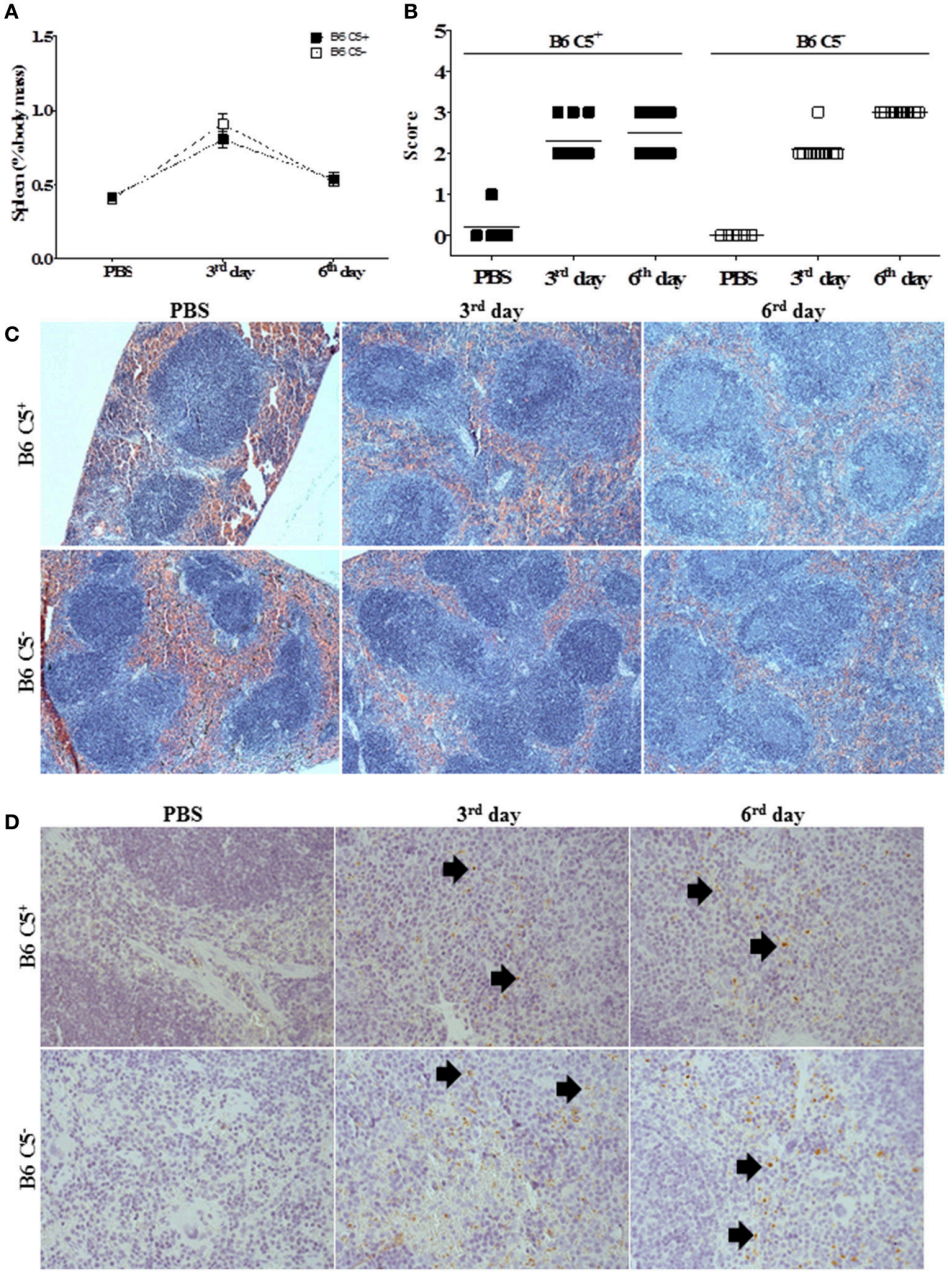
Spleen alterations during LPF infection. Mice were inoculated i/p with 1.5 × 10^8^ LPF or only PBS and then euthanized on the third or sixth day post-infection (*n* ≥ 5). **(A)** Splenomegaly was expressed as percentage of spleen mass to the total body mass. **(B)** Score of morphological alterations. **(C)** Spleen sections stained with HE. **(D)** Immunochemical analysis using anti-leptospiral antibodies. Arrows indicate the presence of LPF antigen.

Both mouse strains developed similar splenomegaly during infection by LPF (Figure 4A). Histopathological analysis indicated that white pulp is expanded after inoculation of LPF, followed by clonal expansion of lymphocytes (Figures 4B,C). On the third day of infection, we observed increased perifolicular activity, indicative of B lymphocyte proliferation. On the sixth day of infection T cell expansion in the centrofolicular region (periarteriolar lymphoid sheath) is more evident. However, no significant differences in the spleen parenchyma could be observed between B6 C5^+/+^ and B6 C5^−/−^ infected mice (Figures 4B,C). The presence of LPF antigens was observed in the spleen of all infected mice (Figure 4D), however no significant differences were observed in this organ at the third and sixth days of infection when B6 C5^+/+^ and B6 C5^−/−^ strains were compared.

### Liver and blood cytokine levels

In spite of a more intense inflammatory response observed in the presence of C5, no significant changes in the concentration of cytokines in the liver of infected mice were observed in B6 C5^+/+^ mice when compared to B6 C5^−/−^ mice (Supplementary Figure 5). The hepatic levels of TNF, IL-6, IL-1β, and IL-12p70 did not change significantly upon infection (Supplementary Figures 5A,B,D,F). B6 C5^−/−^ mice inoculated with PBS presented a higher concentration of liver IL-12p40 than B6 C5^+/+^. On the other hand, B6 C5^−/−^ mice presented a lower concentration of liver IL-12p40 on the sixth day of infection when compared with PBS-treated mice (Supplementary Figure 5E). In the absence of C5, a tendency to lower IL-10 levels were observed on the sixth day of infection (*p* = 0.055) when compared to B6 C5^+/+^ mice (Supplementary Figure 5C) at the same time. Blood levels of TNF-α, IFN-γ, and IL-6 were not significantly affected by LPF infection (Supplementary Figures 5G–I). The concentrations of MCP-1 (CCL2), IL-10, and IL-12p70 in the serum from both B6 C5^+/+^ and B6 C5^+/+^ mice were below detectable levels on the third and the sixth days post-infection.

**Figure 5:**
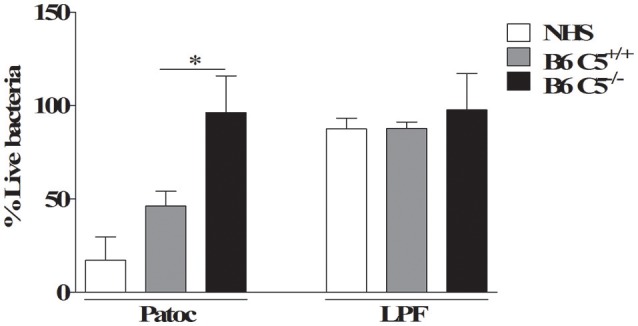
Percentage of viable LPF after *in vitro* incubation with mouse or human serum. 1 × 10^8^ LPF (pathogenic) or non-pathogenic *L. biflexa* sorovar Patoc strain Patoc were incubated for 2 h in 40% of B6 C5^+/+^, B6 C5^−/−^mice serum or normal human serum (NHS) used here as positive control. The number of viable leptospires was counted using dark-field microscopy. The percentage of viable leptospires in the presence of serum was calculated considering respectively heat-inactivated B6 C5^+/+^ mice serum, heat-inactivated B6 C5^−/−^ mice serum or heat-inactivated NHS considered 100% survival (negative controls). ^*^indicates *p* < 0.05.

### LPF is resistant to CS mediated killing by murine serum

To investigate the possible role of C5 in murine resistance to LPF infection, cultures of this pathogenic leptospire were incubated *in vitro* with 40% serum from B6 C5^+/+^, B6 C5^−/−^ mice, or 40% NHS. Since non-pathogenic *L. biflexa* sorovar Patoc strain Patoc is CS-sensitive and is rapidly killed *in vitro* in the presence of NHS (Barbosa et al., 2009), it was included as a positive control. As a negative control, leptospires were also incubated with heat-inactivated serum from B6 C5^+/+^ mice for 2 h before counting the number of viable cells. The number of viable leptospires in the presence of heat-inactivated serum from B6 C5^+/+^ mice was considered 100%. Figure 5 shows that while non-pathogenic leptospires *L. biflexa* survives only in C5 deficient mouse serum, LPF is resistant to *in vitro* lysis mediated by MAC formed in the presence of NHS or serum from both B6 C5^+/+^ and B6 C5^−/−^ mice strains.

## Discussion

One of the first studies of the innate immune response against leptospires pointed to the importance of Toll like receptors (TLR) TLR-2 and TLR-4 to recognize leptospiral pathogen patterns when it was observed that TLR-2 and TLR-4 knockouts rapidly die when infected with this bacterium (Werts et al., 2001). In addition, the role of specific antibodies to control this infection has been studied by several groups (Adler and Faine, 1976; Chassin et al., 2009). (Kobayashi, 2001) observed that guinea pigs, highly susceptible to *Leptospira*, are protected by administering immune serum from convalescent leptospirosis patients. This pointed to the importance of B lymphocytes and antibodies to the acquired immune response in this case.

Since rodents are in general considered resistant to *Leptospira*, we decided to use a murine model to explore the *in vivo* importance of C5 in the first days of infection comparing several parameters in B6 C5^+/+^ and B6 C5^−/−^ mice. All animals (male; 4–6 weeks old) survived at least up to 8 days of infection with 1.5 × 10^8^ LPF. Similar results were observed when we infected another C5 deficient (A/J) mouse strain with the same inoculum. Different results were observed by Ratet et al. (2014) when they infected 7–10 weeks old female wild type C57BL/6J mice with 10^8^ pathogenic *L. interrogans* serovar Manilae. They reported that all animals developed septicemia which led to death on the third day of infection, indicating that even though mice are considered resistant to leptospires, they can be more or less vulnerable to this infection depending on the combination of *Leptospira* serovar pathogenicity and animal characteristics.

In our model, on the third day of infection we detected a higher LPF number in the liver of infected B6 C5^−/−^ mice when compared to the wild type group, indicating that the CS is important to limit bacterial proliferation during the early days of infection. Since the LPF load in liver is controlled on the sixth day in both B6 C5^−/−^ and B6 C5^+/+^ mice, other participants of the innate immune response are acting together. Phagocytic cells such as Kupffer cells could limit the spread of this pathogen in this organ, through Complement receptors (CR) such as CR1, CR3, CR4 (Hinglais et al., 1989), and CRIg (Helmy et al., 2006). It is likely that the release of activated fragments C3b and iC3b, the most important CS opsonins, are generated equally by B6 C5^+/+^ and B6 C5^−/−^ mice after activation of the Alternative or Lectin Pathways. This result could also suggest that LPF could be somehow refractory to lysis by MAC. In agreement with this hypothesis, da Silva et al. (2015) demonstrated that LPF is able to bind to human vitronectin, a soluble regulatory protein that binds to C5b67 (Podack et al., 1984; Singh et al., 2010), and consequently inhibits surface MAC formation. It is worth remembering that leptospiral ligands such as LigA, LigB, and LcpA, present exclusively on the surface of pathogenic leptospires, are capable of binding to host Factor H and C4BP to control CS activation on their surface (Castiblanco-Valencia et al., 2012; da Silva et al., 2015). Considering that non-pathogenic leptospires *L. biflexa* survived when incubated *in vitro* with serum from C5 deficient mice, they could possibly survive during infection *in vivo* in these animals. However, this question remains to be investigated, since other components of innate immunity should contribute to the elimination of this spirochete in the host.

Although the survival of mice was independent of the presence of C5, hepatic lesions were observed on the third and sixth days of infection in higher score in the liver of wild type mice, suggesting that the presence of C5 and its fragments leads to a local inflammatory response. Likewise, an increase in the number of Kupffer cells in the sinusoids and leukocyte infiltrates in the liver was observed as previously reported (Chassin et al., 2009; da Silva et al., 2012). In addition, other lesions such as areas of hepatocyte necrosis and destrabecullation were also observed, although at a lower frequency.

Using C3H/HeJ mice, Chen et al. (2017) concluded that macrophages are the main phagocytic cells (predominating over neutrophils) during infection with *L. interrogans* strain Lai. However, neutrophils may also help to control this infection by releasing extracellular traps (NETs) when in contact with *L. interrogans* serovar Copenhageni strain Fiocruz L1-130 leading to leptospiral killing (Scharrig et al., 2015). When neutrophils were depleted *in vivo* after use of monoclonal antibody mAb1A8, the number of *L. interrogans* increased in the liver on the third day of infection and in the kidney after 14 days of infection, indicating that neutrophils are important in the early days of infection to control leptospirosis. Neutropenia was observed in other study by Stefos et al. (2005) during murine infections with *L. interrogans*. In contrast, in human leptospirosis, the total number of polymorphonuclear neutrophils increases in the first days post-infection (Raffray et al., 2016).

Increased numbers of circulating monocytes have been observed in cases of sepsis in hospitalized leptospirosis patients (Hoser et al., 2012). Leptospires are able to infect human and murine macrophages (Merien et al., 1997) and once internalized, the bacteria may trigger changes in host cell gene expression, leading to apoptosis (Merien et al., 1997; Hu et al., 2013; Xue et al., 2013). The gene expression alterations observed in macrophages from leptospires-infected organisms might also occur in monocytes present in the peripheral circulation, suggesting that programmed cell death may also be occurring in this cell type (Jin et al., 2009; Xue et al., 2013).

C5 protein is important for the activity of different cell types, including lymphocytes, and its absence is responsible for lower lytic activity of T CD8^+^ lymphocytes and reduced cytokine synthesis by T CD4^+^ lymphocytes in different experimental models (Kim et al., 2004; Moulton et al., 2007; Strainic et al., 2008). The interaction of C5a with its receptors present on T CD4^+^ lymphocytes provides the survival stimuli for these cells *in vitro* (Strainic et al., 2008). Although there are no studies that show the participation of C5 in the viability of T CD8^+^ lymphocytes, the activation of these cells is facilitated in the presence of C5a (Strainic et al., 2008). In the case of C5 deficient mice, the lack of C5 may have reduced the stimulation that T CD8^+^ lymphocytes receive to survive and proliferate, resulting in fewer cells in the circulation (Strainic et al., 2008). It has also been shown that the stimulation of T lymphocytes in the presence of C5a reduces the percentage of apoptotic cells and the C5a-C5aR axis stimulates cell expansion (Lalli et al., 2008). The spleen of both C5-deficient and wild-type mouse strains were shown to be a site of much cellular activity during infection. The spleen acts as an active organ in the elimination of circulating leptospires and traces of LPF antigen were found mainly in the red pulp, where macrophages reside.

Finally, it was not possible to observe renal lesions or detect leptospires in the kidneys of the mice in our experimental model through histological and imunochemical analysis. Although the immunohistochemical analysis did not indicate the presence of LPF in the kidneys, the use of more sensitive methods like qPCR would have been better indicated to compare if this leptospiral load would be significantly higher in the absence of C5. Even so, the bacterial load would have been much lower than that observed in the liver. The pathogenesis of acute kidney injury in leptospirosis is the direct nephrotoxic action of the leptospira infection and toxins release, but hemodynamic alteration, jaundice, and rhabdomyolysis (a disruption of skeletal muscle integrity) are also associated (Daher Ede et al., 2010; Abreu et al., 2017). Taking together the biochemical assay results regarding the elevation of AST only in C5^−/−^ mice on the third day post infection and uric acid in both mice on the sixth day post infection, we can suggest that the infection may be inducing rhabdomyolysis in a manner independent of C5. Although the kidney is an important organ for infection because it is a site where leptospires are fixed and later eliminated with the urine, apparently LPF was not able to colonize this organ in the first week of infection. It is possible that the serovar used in our laboratory requires longer periods of time to reach the kidney. Chassin et al. (2009) also observed relatively low leptospiral load on the third day of infection in the kidney and lung from wild type C57Bl/6J mice infected with pathogenic *L. interrogans* serovar Copenhageni strain Fiocruz LI-130.

Our results suggest that C5 may play a role in the direct killing of LPF only up to the third day of infection *in vivo*. Considering the inflammatory properties of the C5a fragment, the presence of C5 is associated with tissue lesions observed in the liver of mice infected with *L. interrogans*. However, this variation did not significantly alter the ability of mice to control infection caused by LPF, suggesting a minor role for C5 in this infection model. The possibility that C3b/iC3b opsonins play a more important role than the MAC (C5b-9_n_) or the fragment C5a to control leptospiral murine infection remains to be further investigated.

## Ethics statement

This study was carried out in accordance with the recommendations of Ethics Committee on Animal Experimentation of Institute of Biomedical Sciences from the University of São Paulo. The protocol was approved according to the Certificate 061/10/CEEA.

## Author contributions

IdC and LB did the experiments; TF, LB, and AG-M helped with the leptospires cultures and counting viable leptospires; MA determined the serum concentrations of cytokines; AdS helped with immunochemistry analyses, SV provided the cultures and confirmed the pathogenicity of leptospires, IdC, LB, and LI designed the experiments, analyzed the results and wrote the manuscript.

### Conflict of interest statement

The authors declare that the research was conducted in the absence of any commercial or financial relationships that could be construed as a potential conflict of interest.

## Supplementary material

Supplementary Figure 1 Immunohistochemical analysis of liver from infected mice. Mice were inoculated i/p with 1.5 × 10^8^ LPF or PBS and then euthanized on the third or the sixth day, when the liver was collected for LPF antigen analysis (*n* ≥ 5). The arrows indicate the presence of labeled LPF antigens.

Supplementary Figure 2 Serum concentrations of hepatic ALT **(A)** and AST **(B)** enzymes in infected mice. Mice were inoculated i/p with 1.5 × 10^8^ LPF or PBS and then euthanized on the third or the sixth day, when the blood was collected for analysis (*n* ≥ 5). ALT and AST were used as indicators of liver damage. The significant differences (*p* < 0.05) are represented as follows: ^b^vs. third day and ^c^vs. B6 C5^+/+^.

Supplementary Figure 3 Histopathological analysis of lung from infected mice. Mice were inoculated i/p with 1.5 × 10^8^ LPF or PBS and then euthanized on the third or the sixth day, when the lung was collected for histopathological analysis (*n* ≥ 5). **(A)** The liver sections (3–5 micrometers) were stained with HE and evaluated at 200x magnification. **(B)** Scores of histopathological alterations.

Supplementary Figure 4 Histopathological analysis of kidney from LPF infected mice. Mice were inoculated i/p with 1.5 × 10^8^ LPF or PBS and then euthanized on the third or the sixth days, when the kidney was collected for histopathological analysis (*n* ≥ 5). **(A)** The kidney sections (3–5 micrometers) were stained with HE, 200x magnification. (**B**) Serum levels of urea and uric acid. The significant differences (*p* < 0.05) are represented as follows: ^a^vs. PBS; ^b^vs. third day.

Supplementary Figure 5 Concentrations of pro- and anti-inflammatory cytokines during infection by LPF. B6 C5^+/+^ and B6 C5^−/−^ mice were inoculated i/p with 1.5 × 10^8^ LPF or PBS and then euthanized on the third or the sixth day, when the liver was collected and prepared for cytokine concentration determination (*n* ≥ 5). Liver cells were disrupted and treated with protease inhibitors. The concentration of several cytokines **(A–F)** was analyzed in the liver extracts by ELISA (normalized with respect to total protein) or in the serum of infected mice (*n* ≥ 3) by CBA **(G–I)**. un: undetected. The significant differences (*p* < 0.05) are represented as follows: ^a^vs. PBS; ^c^B6 C5^+/+^vs. B6 C5^−/−^.
