# Supplementary figures and images for: Role of Murine Complement Component C5 in Acute in Vivo Infection by Pathogenic Leptospira interrogans
Source: Front Cell Infect Microbiol. 2018 Mar 8;8:63. doi: 10.3389/fcimb.2018.00063 (PMC5852101; doi:10.3389/fcimb.2018.00063)

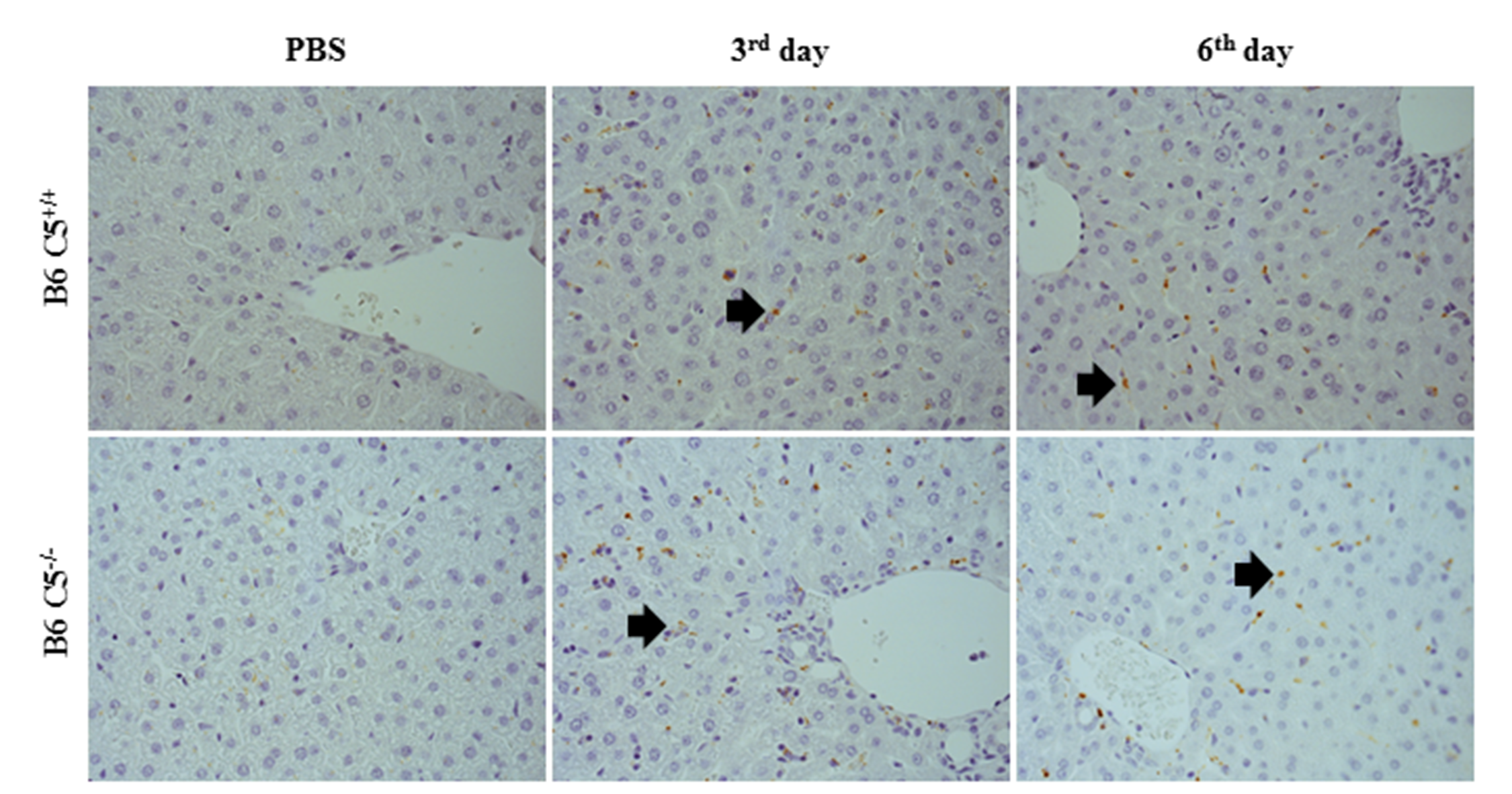

Supplement: Supplementary Figure 1 — Immunohistochemical analysis of liver from infected mice. Mice were inoculated i/p with 1.5 × 10^8^ LPF or PBS and then euthanized on the third or the sixth day, when the liver was collected for LPF antigen analysis (n ≥ 5). The arrows indicate the presence of labeled LPF antigens. [file Image1.tif]

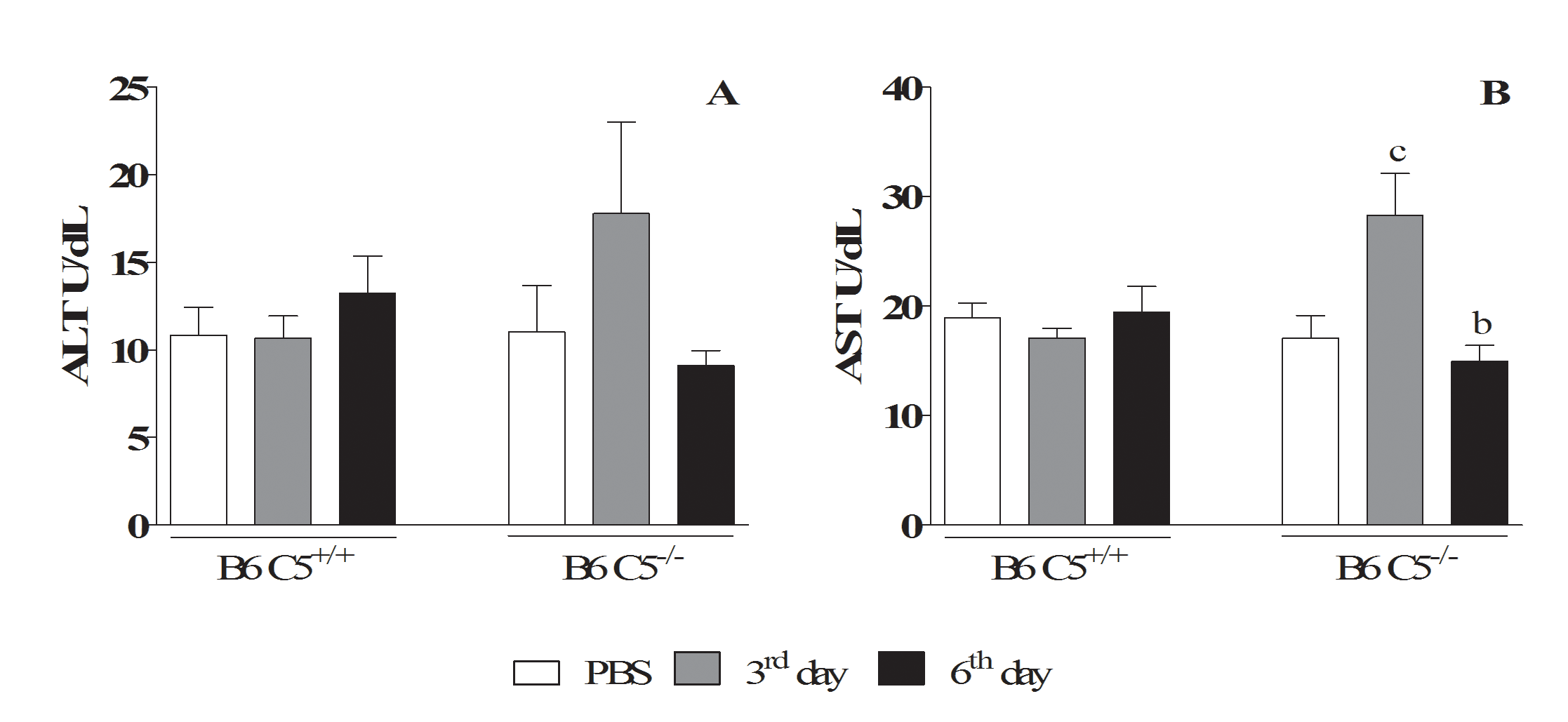

Supplement: Supplementary Figure 2 — Serum concentrations of hepatic ALT (A) and AST (B) enzymes in infected mice. Mice were inoculated i/p with 1.5 × 10^8^ LPF or PBS and then euthanized on the third or the sixth day, when the blood was collected for analysis (n ≥ 5). ALT and AST were used as indicators of liver damage. The significant differences (p < 0.05) are represented as follows: bvs. third day and cvs. B6 C5+/+. [file Image2.TIF]

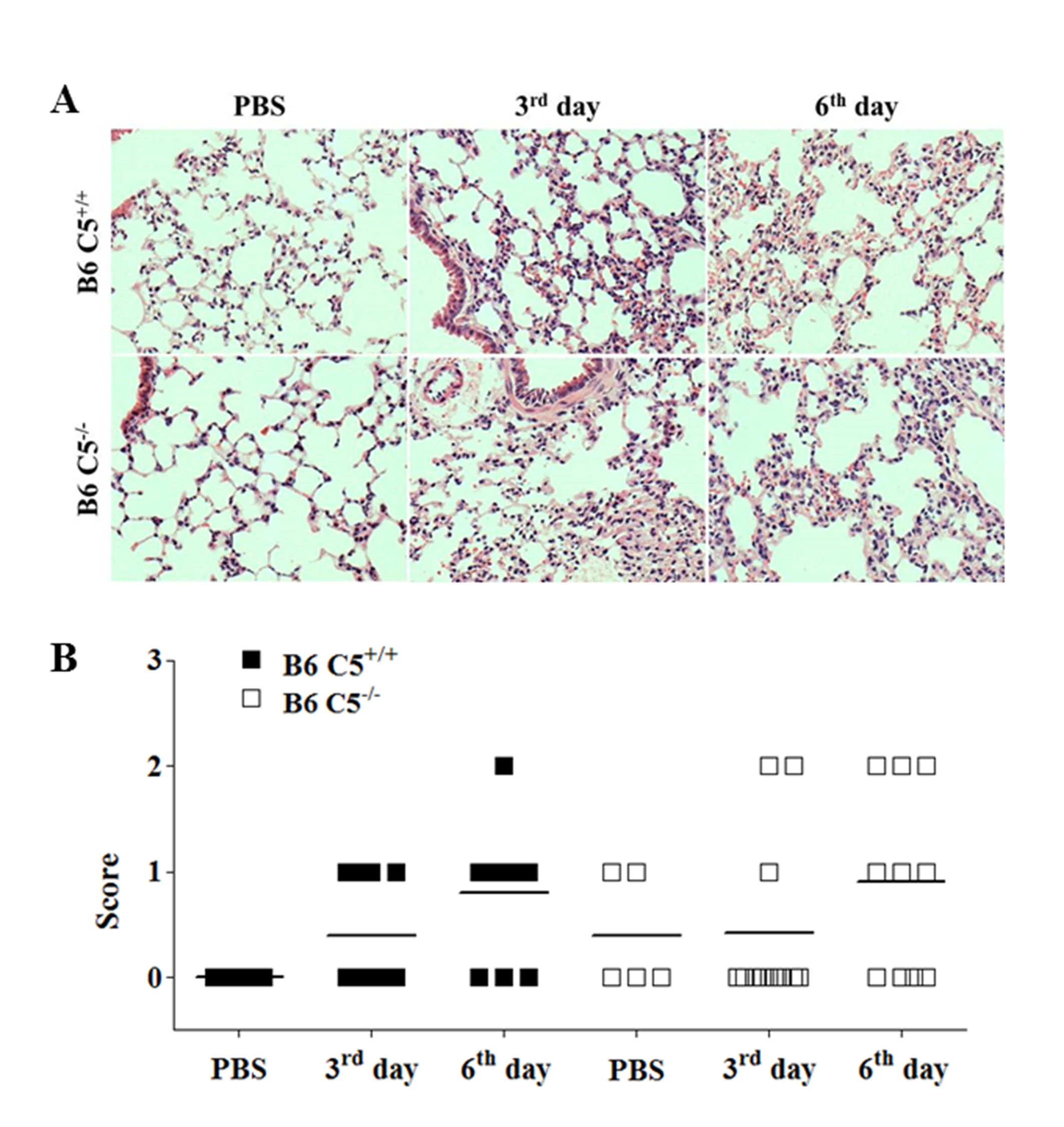

Supplement: Supplementary Figure 3 — Histopathological analysis of lung from infected mice. Mice were inoculated i/p with 1.5 × 10^8^ LPF or PBS and then euthanized on the third or the sixth day, when the lung was collected for histopathological analysis (n ≥ 5). (A) The liver sections (3–5 micrometers) were stained with HE and evaluated at 200x magnification. (B) Scores of histopathological alterations. [file Image3.tif]

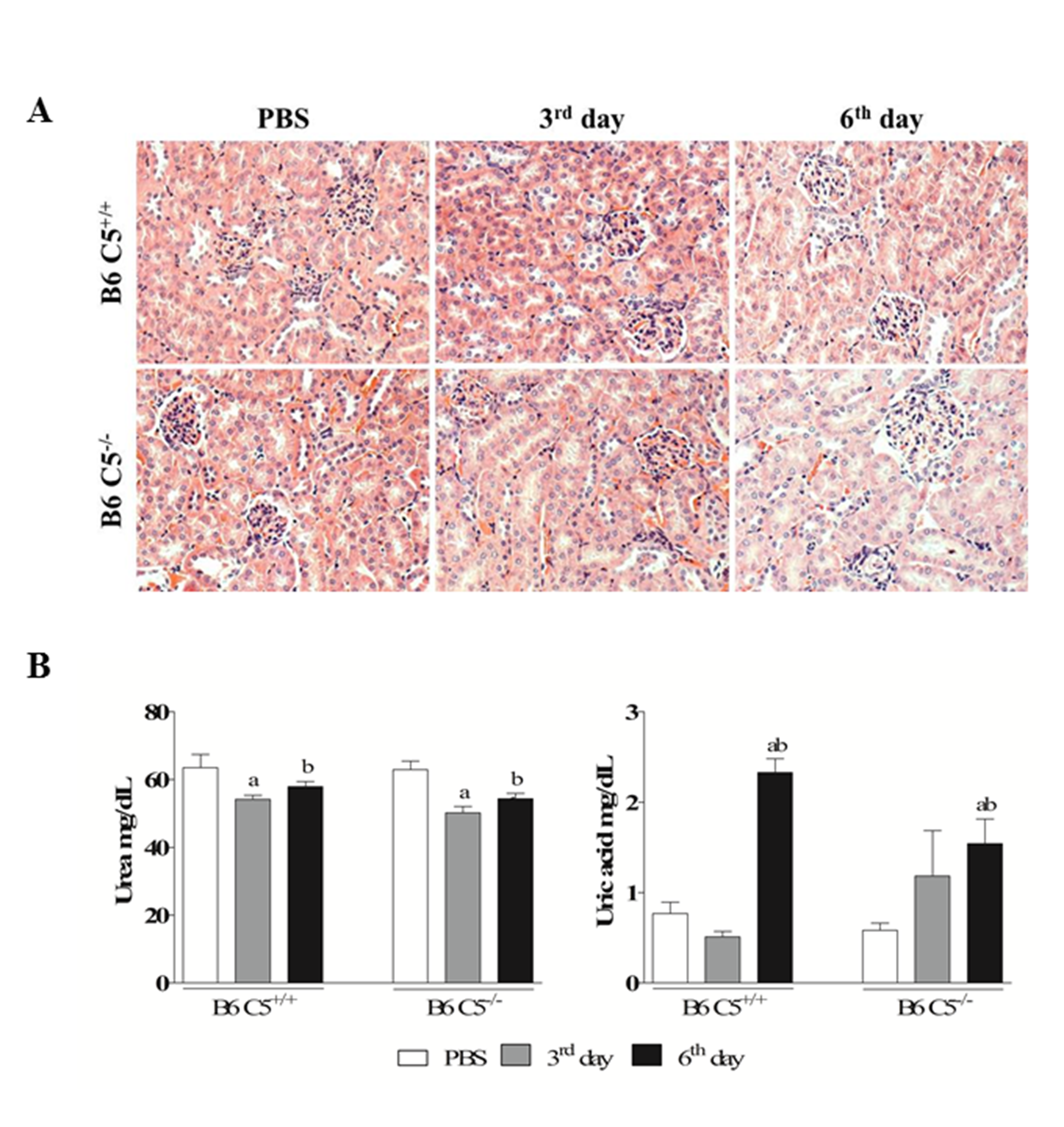

Supplement: Supplementary Figure 4 — Histopathological analysis of kidney from LPF infected mice. Mice were inoculated i/p with 1.5 × 10^8^ LPF or PBS and then euthanized on the third or the sixth days, when the kidney was collected for histopathological analysis (n ≥ 5). (A) The kidney sections (3–5 micrometers) were stained with HE, 200x magnification. (B) Serum levels of urea and uric acid. The significant differences (p < 0.05) are represented as follows: avs. PBS; bvs. third day. [file Image4.TIF]

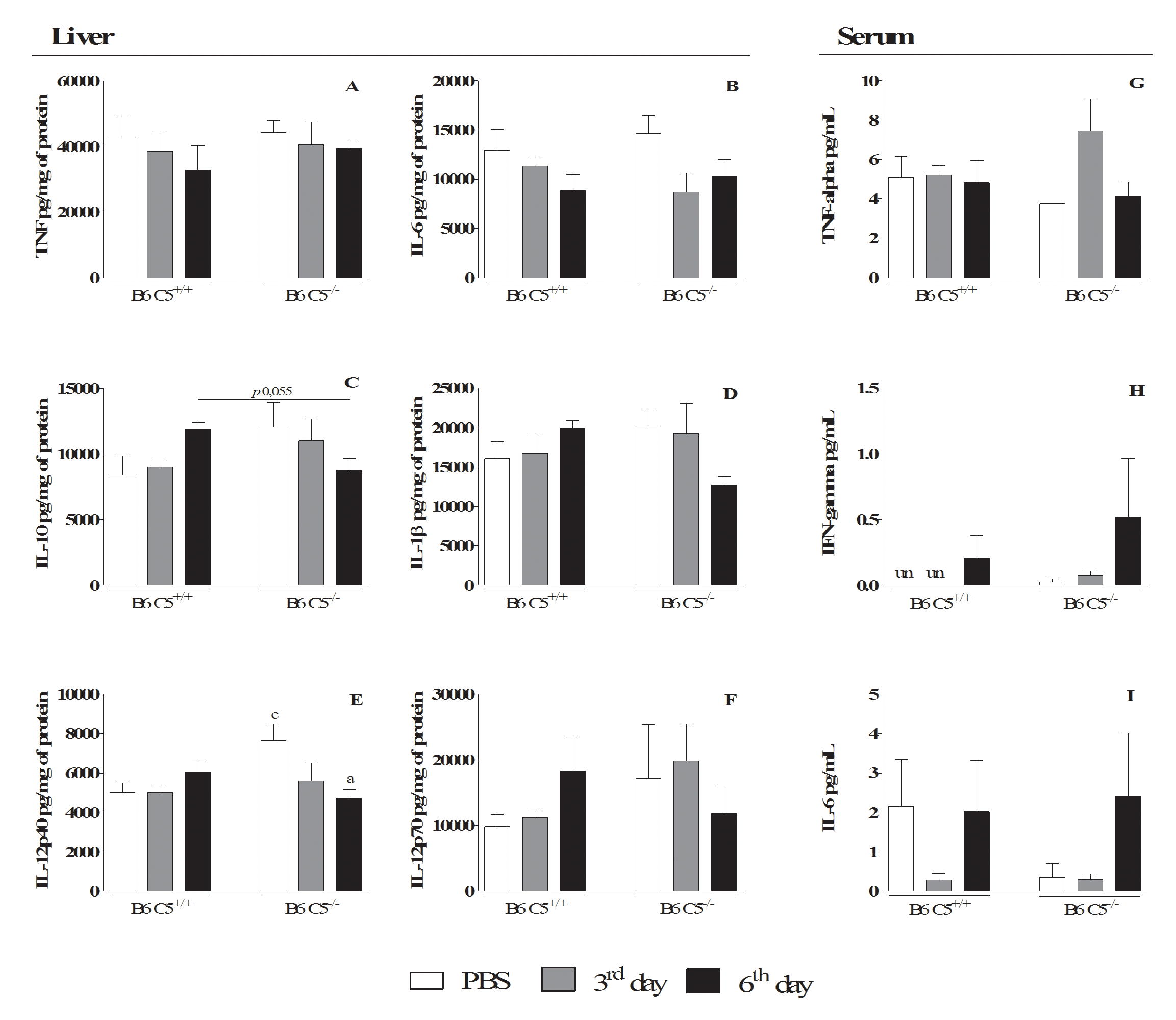

Supplement: Supplementary Figure 5 — Concentrations of pro- and anti-inflammatory cytokines during infection by LPF. B6 C5+/+ and B6 C5−/− mice were inoculated i/p with 1.5 × 10^8^ LPF or PBS and then euthanized on the third or the sixth day, when the liver was collected and prepared for cytokine concentration determination (n ≥ 5). Liver cells were disrupted and treated with protease inhibitors. The concentration of several cytokines (A–F) was analyzed in the liver extracts by ELISA (normalized with respect to total protein) or in the serum of infected mice (n ≥ 3) by CBA (G–I). un: undetected. The significant differences (p < 0.05) are represented as follows: avs. PBS; cB6 C5+/+vs. B6 C5−/−. [file Image5.TIF]
